# Preclinical SC and IV repeat-dose toxicology of a cowpea mosaic virus – A cancer immunotherapy candidate

**DOI:** 10.1016/j.toxrep.2025.102022

**Published:** 2025-04-07

**Authors:** Stephan T. Stern, Jessica Fernanda Affonso de Oliveira, Jamie Gatus, Elijah Edmondson, Barry W. Neun, Marina A. Dobrovolskaia, Nicole F. Steinmetz

**Affiliations:** a Nanotechnology Characterization Lab, Cancer Research Technology Program, Frederick National Laboratory for Cancer Research sponsored by the National Cancer Institute, Frederick, MD 21702, USA; b Aiiso Yufeng Li Family Department of Chemical and Nano Engineering, University of California San Diego, 9500 Gilman Dr., La Jolla, La Jolla, CA 92093, USA; c Shu and K.C. Chien and Peter Farrell Collaboratory, University of California San Diego, 9500 Gilman Dr., La Jolla, CA 92093, USA; d Center for Nano-ImmunoEngineering, University of California San Diego, 9500 Gilman Dr., La Jolla, CA 92093, USA; e Department of Bioengineering, University of California San Diego, 9500 Gilman Dr., La Jolla, CA 92093, USA; f Molecular Histopathology Laboratory, Laboratory of Animal Sciences Program, Frederick National Laboratory for Cancer Research sponsored by the National Cancer Institute, 8560 Progress Drive, Frederick, MD 21701, USA; g Department of Radiology, University of California San Diego, 9500 Gilman Dr., La Jolla, CA 92093, USA; h Institute for Materials Discovery and Design, University of California San Diego, 9500 Gilman Dr., La Jolla, CA 92093, USA; i Moores Cancer Center, University of California San Diego, 9500 Gilman Dr., La Jolla, CA 92093, USA; j Center for Engineering in Cancer, Institute of Engineering Medicine, University of California San Diego, 9500 Gilman Dr., La Jolla, CA 92093, USA

**Keywords:** Plant viruses, Cowpea mosaic virus (CPMV), Pharmacology, Toxicology

## Abstract

Cancer immunotherapies focus on boosting the immune system to recognize and eliminate tumor cells. Amongst the various biologics under development for cancer immunotherapy, our team has focused on the study of plant viruses in this context. We have shown that intratumoral administration of cowpea mosaic virus (CPMV) relieves the immunosuppressive tumor microenvironment and elicits a potent, systemic and durable anti-tumor immune response. The potency of CPMV has been demonstrated in several tumor mouse models and in companion canine cancer patients. Toward clinical development, we here studied the pharmacology and safety of CPMV. The repeat-dose toxicity of CPMV was evaluated in female Sprague Dawley rats. Rats received three weekly treatments (subcutaneous or intravenous) of a fixed dose (∼20 mg/kg), and complete necropsy was performed either 24 hrs (acute toxicity group) or 14 day (recovery group) post-dose. All animals reached the scheduled euthanasia times, and no clinical abnormalities were noted during the study period. Important clinical chemistry, hematology and histopathology findings included decreased albumin/globulin ratio, leukocytosis, neutrophilia, monocytosis, and lymphoid hyperplasia (Dunnett’s test, p<0.05) – these changes support the immunostimulatory mode of action for CPMV. All other changes were considered mild, within historical range for the model, and/or not biologically significant. Neither a maximum tolerated dose (MTD) nor a no-observable adverse effect level (NOAEL) was established in this study. Overall, data indicate a good safety profile for the CPMV cancer immunotherapy candidate.

## Highlights

• No deaths or abnormal clinical signs were observed during the study.
• Immune activation markers support CPMV's immunostimulatory mechanism.
• Other findings were mild, within historical range, or not biologically significant.
• No MTD or NOAEL was established during the repeat-dose toxicity study.

## Graphical Abstract

**Figure:**
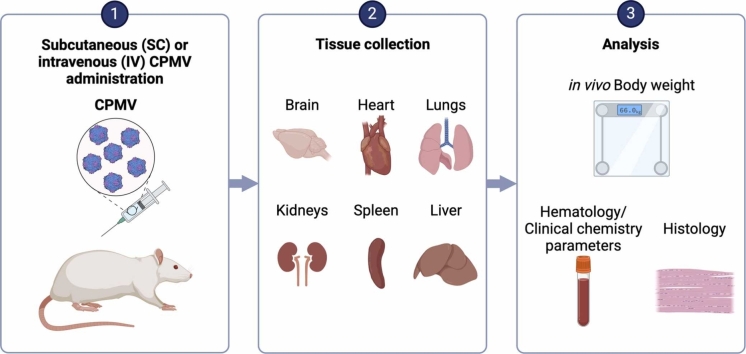

## 1 Introduction

Cancer remains a leading cause of morbidity and mortality worldwide, with solid tumors such as melanoma, breast, colorectal, prostate, ovarian cancer accounting for most cases. According to the World Health Organization (WHO), global cancer incidence continues to rise, with an estimated 20 million new cases and 10 million deaths in 2022. Solid tumors pose significant treatment challenges due to their immunosuppressive microenvironment and resistance to systemic therapies. Intratumoral immunotherapy is an emerging approach designed to overcome these challenges by directly injecting immunostimulatory agents into tumors, enhancing local and systemic anti-tumor responses [1]. The intratumoral immunotherapy market is rapidly expanding, with an estimated CAGR exceeding 10 %, driven by novel biologics, combination strategies, and growing clinical validation. As innovation accelerates, intratumoral therapies have the potential to transform cancer treatment by improving efficacy while reducing systemic toxicity.

Cancer immunotherapies (systemic or intratumorally applied) harness the patient’s immune system to recognize and eradicate tumor cells [2]. The US Food and Drug Administration (FDA) has already approved several cancer immunotherapies and many promising approaches are in the development pipeline, including various immune checkpoint inhibitors (e.g., CTL4, PDL1-PD1), tumor vaccines (Sipuleucel-T), cell-based immunotherapies (CAR-T), and small molecule-based immunotherapies (STING agonists) [3]. Furthermore, oncolytic viruses engineered to lyse tumor cells and express cytokines show promise as intratumoral immunotherapies [4], a bacteriophage-based nanoparticle delivering small molecule toll-like receptor (TLR) 9 agonists is undergoing clinical testing (drug candidate: Vidutolimod, SD-101) [5], and finally we and others are developing various plant virus-based nanotechnologies as cancer immunotherapies [6].

Specifically, we have focused on the translational development of cowpea mosaic virus (CPMV) as intratumoral immunotherapy. Intratumoral CPMV stimulates potent, systemic and durable anti-tumor immunity in murine tumor models [7], [8], [9], [10], [11], [12], [13] and in canine cancer patients (companion pets) with spontaneous tumors [14], [15], [16]. CPMV activates innate immune cells (myeloid and professional antigen-presenting cells) and overcomes immunosuppression within the tumor microenvironment (TME), launching both local and systemic adaptive anti-tumor immunity (lymphocytes) to suppresses both local and distant metastases, while inducing immune memory that prevents recurrence [2], [17]. It is important to understand that CPMV cancer immunotherapy is conceptually distinct from oncolytic virus therapy: oncolytic viruses function by infecting and killing cancer cells – however, CPMV targets innate immune cells to prime systemic anti-immunity. A particular advantage is – because CPMV targets the innate immune system – virus-specific antibodies (which may be formed during repeat treatment schedules) are not neutralizing but boost the anti-tumor response [18]. While effective as monotherapy, CPMV intratumoral immunotherapy can also improve efficacy of systemic immunotherapy with checkpoint blocking antibodies by expanding the pool of anti-tumor T cells that respond to checkpoint blockade [9], [11], [19]. Given the anti-tumor potency observed in mouse tumor models and canine patients, we seek to investigate the pharmacology and toxicology of CPMV to drive its clinical development strategy.

CPMV is a positive-sense, bipartite, single-stranded RNA virus [20], [21] that forms icosahedral, 30 nm-sized particles with its structure known to near atomic resolution [17]. The virions are formed by 60 copies of two different types of coat proteins, the small (S, 213 amino acids) subunit and the large (L, 374 amino acids) subunit arranged in *pseudo T = 3* symmetry [17], [22]. CPMV is from the family Secoviridae, genus Comovirus and plant picornavirus. CPMV is infectious toward plants and has a rather narrow natural host-range, it normally infects legumes and was first reported in *Vigna unguiculata*. CPMV is transmitted by leaf-feeding beetles, thrips and grasshoppers. In addition to the natural hosts, species from several families – including legumes and *Nicotiana benthamiana* – are known to be susceptible to the virus and transmission can be achieved experimentally by mechanical inoculation. In systemic infected plants CPMV typically causes mosaic or mottling symptoms [23].

In terms of pharmacology and toxicology, CPMV is infectious toward plants, but there are no reports indicating infection in mammals [24]. Others reported the pharmacology of CPMV in mice after single-dose intravenous (i.v.) administration [25]: CPMV has a short plasma half-life (∼20–30 mins) and the majority of CPMV is cleared by the liver and spleen. At an i.v. dose of CPMV of up to 100 mg/kg body weight, hematology was reported normal. Histology showed elevated B lymphocytes in the spleen, but there were no signs of tissue degeneration or necrosis in any major organs. The study concluded that CPMV appears to be a safe and non-toxic at doses up to 100 mg per kg of body weight (for reference, in our canine cancer trials we dosed CPMV twice intratumorally at ∼0.01 mg/kg body weight). This is consistent with studies demonstrating hemocompatibility and immunocompatibility of CPMV using human whole blood and its derivatives [24]. Biodistribution was also reported after intratumoral (i.t.) dosing using the B16F10 dermal melanoma model. Given its nanoparticle character, most of the i.t. dose was retained in the tumor with minimal leaching -- CPMV particles that leached showed broad biodistribution. Data suggest clearance via the mononuclear phagocytic system followed by biliary excretion. Shedding of infectious particles was not apparent [26].

As a next step, we here report the results of a female Spargue Dawley rat repeat-dose toxicity study. Rats received three weekly doses of 20 mg CPMV/kg, dosing i.v. or s.c., with complete necropsy 24 h (acute toxicity group) and 14 days (recovery group) post-dose. Study endpoints included gross description, body and organ weights, hematology, clinical chemistry, and histopathology of select organs. Experiments were carried according to the NCL Assay Cascade (https://www.cancer.gov/nano/research/ncl/assay-cascade).

## 2 Materials and methods

### 2.1 Preparation and characterization of cowpea mosaic virus (CPMV) nanoparticles

CPMV nanoparticles were isolated from mechanically inoculated black-eyed pea plants (*Vigna unguiculata* no. 5 from Morgan County Seeds, Lot SW211211075B, Cat #203) and purified following protocols previously published [27]. To minimize endotoxin contamination, all buffers and glassware were autoclaved, and the glassware was additionally baked at 200°C for four hours before use. Eight batches of CPMV were prepared pooled for the toxicology study.

For CPMV characterization, we followed our previously described protocols [24], [28]. In brief, CPMV nanoparticle concentration was determined using a NanoDrop 2000 spectrophotometer (Thermo Fisher Scientific) and Beer-Lambert law (A_260_ = εcl; where CPMV ε_260_nm = 8.1 mL.mg^−1^ cm^−1^ and l = 0.1 cm). Sizing and purity were analyzed using size exclusion chromatography (SEC), dynamic light scattering (DLS), and transmission electron microscopy (TEM). For SEC, an ÄKTA-Pure fast protein liquid chromatography system (GE Healthcare LifeSciences) equipped with Superose 6 Increase 100 GL column was used and CPMV was analyzed at 0.5 mg.mL^−1^ and a flow rate of 0.5 mL.min^−1^. The hydrodynamic diameter of CPMV (at 0.2 mg.mL^−1^) was measured using DLS and a Zetasizer Nano ZSP/Zen5600 (Malvern Panalytical). For TEM imaging, CPMV (0.2 mg.mL^−1^ diluted in deionized water) was loaded onto discharged, formvar carbon film-coated, 400-mesh hexagonal copper grids (Electron Microscopy Sciences), washed, and then stained with 2 % (w/v) uranyl acetate. Samples were imaged using JEOL 1400Plus Transmission Electron Microscope (Peabody), and images were collected at 30,000x magnification and 80 kV. Finally, CPMV preparations were analyzed by NuPAGE and agarose gel electrophoresis: In brief, for NuPAGE analysis of the CPMV coat proteins, CPMV (12 µg) mixed with 4x lithium dodecyl sulfate sample buffer and 10X NuPAGE sample reducing agent (Invitrogen) were denatured by heat and then analyzed on precast NuPAGE 4–12 % Bis-Tris Protein Gel and run at 200 V for 40 min using 1x NuPAGE MOPS buffer (all reagents from Thermo Fisher Scientific). Proteins were stained using with GelCode Blue Safe Protein Stain (Thermo Fisher Scientific) and were visualized under white light using ProteinSimple FluorChem R system. For agarose gel electrophoresis of intact CPMV virions, CPMV mixed with 6x Gel Loading Purple Dye (New England Biolabs) was analyzed on 1.2 % (w/v) agarose gels (run at 80 V, 300 mA, 40 min, in 10 mM KP buffer pH 7.0) stained with GelRed Nucleic Acid Gel Stain (Biotium). Gels were imaged using ProteinSimple FluorChem R system under UV light to visualize the RNA, followed by staining with 0.25 % (w/v) Coomassie Brilliant Blue G-250 and then imaged under white light to visualize the protein.

### 2.2 Sterility, endotoxin & β-glucans quantification

Sterility testing was performed following NCL protocol STE-2.4 (https://www.cancer.gov/nano/research/ncl/protocols-capabilities). In brief, samples were plated onto Tryptic Soy (Alpha Teknova, Inc., Hollister, CA, USA) agar plates at several dilutions (stock, 10-, 100- and 1000-fold dilutions of the stock at 1–10 mg.mL^−1^) and allowed to incubate at 37°C for 72 h. The plates were then visually inspected for colony formation. In addition, 100 μL of the stock was spiked into 5 mL of unsupplemented Roswell Park Memorial Institute (RPMI, Hyclone, Cytiva Global Life Sciences Solutions, Marlborough, M, USA) medium and incubated at 37°C for 7 days to detect the presence of autotrophic and/or oligotrophic bacteria. The flasks were then visually inspected to assess turbidity/bacterial growth. To quantify endotoxin, NCL protocols for kinetic chromogenic and/or turbidity Limulus Amebocyte Lysate (LAL) were used (STE-1.4 and STE-1.2, respectively, https://www.cancer.gov/nano/research/ncl/protocols-capabilities). Evaluation of levels of β-glucans in the CPMV was performed following NCL Protocol STE-4 (https://www.cancer.gov/nano/research/ncl/protocols-capabilities) using multiple dilutions (5-, 50- and 500-fold) from the stock concentration (1–10 mg.mL^−1^).

### 2.3 *In vivo* toxicology study

Animal care was provided in accordance with the procedures outlined in the Guide for Care and Use of Laboratory Animals (National Research Council, 1996; National Academy Press, Washington, D.C.). NCI-Frederick is accredited by AAALAC International and follows the Public Health Service Policy for the Care and Use of Laboratory Animals (Health Research Extension Act of 1985, Public Law 99–158, 1986). All animal protocols were approved by the NCI at Fredrick institutional Animal Care and Use Committee (IACUC). This animal study was approved by the IACUC under ASP 22–274.

Female Sprague Dawley (10 weeks-old) were acclimated to the study environment for one week prior to initiation of drug (here CPMV) treatment. Females have been shown to be more sensitive to viral infection- and viral vaccine-induced innate, humoral and cellular immune responses, which could result in CARPA, anaphylaxis, and/or cytokine storm, the anticipated dose-limiting toxicities for CPMV (DOI: 10.4415/ANN_16_02_11). Therefore, as this is a preliminary study in advance of IND-enabling studies, and in consideration of the 3 R’s, we chose to only use the most sensitive animal sex, female. Animal rooms were kept at 50 % relative humidity, 20–22˚C with 12 h light/dark cycles. Rats were housed by treatment group, with two animals/cage (Rat polycarbonate cage type), with ¼” corncob bedding. Animals were allowed ad libitum access to Purina 18 % NIH Block and chlorinated tap water. Animals were randomized to treatment groups based on body weight prior to dosing (group means and statistics resulting from the body weight randomization can be found in the Table S1).

For the *in vivo* toxicology experiments seven animals were used per treatment group (n = 7). Animals were treated with a fixed 5 mg CPMV dose administered in a 500 µL volume, once a week for 3 weeks, administered either subcutaneously (s.c.) or intravenously (i.v.) by tail vein. A q3w x 3 dosing schedule was utilized to mimic the intended clinical regimen and subsequent IND-enabling toxicity studies, that would allow for market authorization under FDA S9 guidelines for cancer pharmaceutical (Guidance for Industry S9 Nonclinical Evaluation for Anticancer Pharmaceuticals, U.S. Department of Health and Human Services Food and Drug Administration, Center for Drug Evaluation and Research (CDER), Center for Biologics Evaluation and Research (CBER), March 2010 ICH, Guidance for Industry Microsoft Word - 9125fnl.doc, accessed 3/11/2025).

Vehicle (0.1 M potassium phosphate buffer pH 7.0) and untreated control groups were also included (Table 1, Table 2). Animal body weights were measured on alternate days (Mondays, Wednesday and Fridays), and animal behavior was monitored daily for signs of morbidity, ≥ 20 % body weight loss, or inability to obtain food or water due to treatment. Animals were euthanized by CO_2_ asphyxiation either 1- or 14-days post dose, for main and recovery groups respectively. A complete necropsy was performed with gross description and weighing of all standard tissues. All tissues collected at necropsy were placed into 10 % (v/v) neutral buffered formalin (NBF) until further analysis. Blood was collected at necropsy by thoracic aorta cut down for full hematology and clinical chemistry panels. *NB:* Random glucose measurements were made, animals were not fasted prior to measurement. Select tissues identified by gross, hematological, or clinical chemistry abnormalities (spleen, liver, lymph node, and femur bone marrow) were evaluated histopathologically by a board-certified veterinary pathologist.

**Table 1:** Study design.

**Group ID**	**# of** **Animals**	**Sex** **(M/F)**	**Treatment**	**Dose Level**	**Dose Volume**	**Vehicle**	**Route**
1	7	F	Vehicle (Main group)	Once a week/3x	500 µL (fixed)	KP buffer	s.c.
2	7	F	Vehicle (Recovery group)		500 µL (fixed)	KP buffer	s.c.
3	7	F	Vehicle (Main group)		500 µL (fixed)	KP buffer	i.v.
4	7	F	Vehicle (Recovery group)		500 µL (fixed)	KP buffer	i.v.
5	7	F	5 mg (∼20 mg/kg) CPMV (Main group)		500 µL (fixed)	KP buffer	s.c.
6	7	F	5 mg (∼20 mg/kg) CPMV (Recovery group)		500 µL (fixed)	KP buffer	s.c.
7	7	F	5 mg (∼20 mg/kg) CPMV (Main group)		500 µL (fixed)	KP buffer	i.v.
8	7	F	5 mg (∼20 mg/kg) CPMV (Recovery group)		500 µL (fixed)	KP buffer	i.v.
9	7	F	Untreated (Main group)	N/A	N/A	N/A	N/A
10	7	F	Untreated (Recovery group)	N/A	N/A	N/A	N/A

**Table 2:** Dose and euthanasia days.

		Dosing (Once a week/3x)		Main Group Euthanasia (MHL, 24 h main)		Recovery Group Euthanasia MHL (14 days recovery)	
Group ID	# of Rats	Tuesday	Wednesday	Wednesday	Thursday	Tuesday	Wednesday
1	7	4	3	4	3		
2	7	4	3			4	3
3	7	4	3	4	3		
4	7	4	3			4	3
5	7	4	3	4	3		
6	7	4	3			4	3
7	7	4	3	4	3		
8	7	4	3			4	3
9	7			4	3		
10	7					4	3
**Total rats to be dosed**		32	24				
Total Rats transfer to MHL				20	15	20	15

### 2.4 Tissues evaluated by histopathology

The predetermined list of tissues that were collected for potential histopathological evaluation included: adrenal, brain, cecum, colon, duodenum, epididymis, esophagus, eye, femoral marrow, femur, gall bladder, Harderian gland, heart, ileum, jejunum, kidney, liver, lung, mammary gland, mandibular lymph node, mesenteric lymph node, nasal sections, ovary, pancreas, parathyroid, pituitary, rectum, salivary gland, skin/subcutis, spinal cord, spleen, stomach, thymus, thyroid, tongue, trachea, urinary bladder, uterus, and vertebra. Any additional tissues with gross findings at necropsy were also collected.

### 2.5 Hematology parameters

Hematology parameters analyzed included differential leukocyte count (basophils, eosinophils, lymphocytes, monocytes and neutrophils), erythrocyte count (RBC), hematocrit (HCT), hemoglobin (Hb), mean corpuscular hemoglobin (MCH), mean corpuscular hemoglobin concentration (MCHC), mean corpuscular volume (MCV), mean platelet volume (MPV), platelet count (PLT), red blood cell distribution width (RDW), and total leukocyte count (WBC), reticulocytes (Retics).

### 2.6 Statistical methods

Statistical analyses were conducted using the software Statistica version 14.1.0.8 (Cloud Software Group, Inc., Palo Alto, CA). Statistical differences for parametric data were determined by ANOVA, with post-hoc comparisons by Dunnett’s test (p<0.05). Nonparametric data were analyzed by the Kruskal-Wallis ANOVA with multiple comparisons test (p<0.05).

## 3 Results and discussion

### 3.1 Characterization of CPMV

**Fig. 1:**
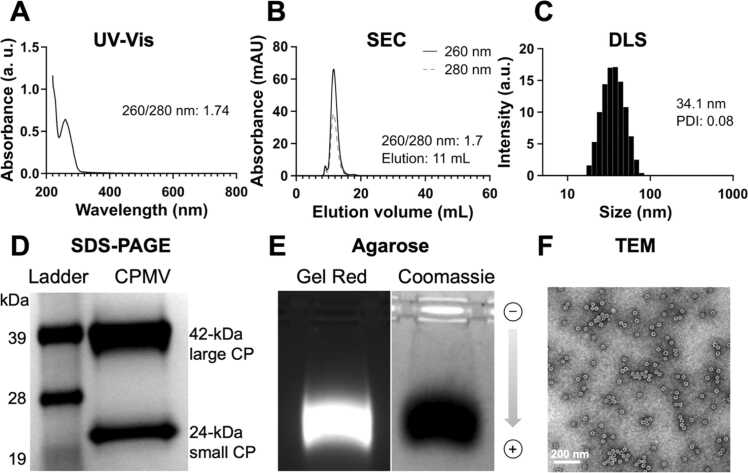
CPMV characterization. (A) Ultraviolet visible spectroscopy (UV-Vis) spectrum. (B) Size exclusion chromatography (SEC) profile using a Superose 6 Increase column. (C) Dynamic light scattering (DLS). (D) Denaturing NuPAGE to detect the 24-kDa small and 42-kDa large coat proteins (CP); SeeBlue™ Plus2 Pre-stained Protein Standard was used as ladder. (E) Native agarose gel electrophoresis verified the co-localization of CPMV’s RNA (Gel Red RNA stain and visualization under UV light) and coat proteins (Coomassie staining and visualization under white light). (F) Transmission electron microscopy (TEM) of negatively-stained CPMV. Graphs were plotted using GraphPad Prism v10.2.0. Data shown in Fig. 1 is representative for all prepared CPMV batches.

To determine CPMV particle integrity and purity a combination of characterization methods were used, as summarized in Fig. 1. Ultraviolet visible spectroscopy (UV-Vis) was used to determine the concentration of CPMV; the A_260/280_ ratio of 1.74 also indicated pure CPMV preparations (Fig. 1A) [29]. This result was also supported by size exclusion chromatography (SEC) with a calculated A_260/280_ ratio of 1.7 (Fig. 1B); CPMV showed the characteristic elution profile eluting between 11–12 mL from the Superose 6 Increase column; broken particles or contaminants were not apparent. Dynamic light scattering (DLS) showed the typical size profile and monodispersity of CPMV (D=34.1 nm with a PDI of 0.08, Fig. 1C). Denaturing gel electrophoresis was used to detect the CPMV’s 24-kDa small and 42-kDa large coat proteins (Fig. 1D). Native gel electrophoresis was used to verify the co-migration of the RNA and CPMV coat proteins, thereby also confirming these particles were intact (Fig. 1E). Finally, in agreement with SEC and DLS data, transmission electron microscopy (TEM) confirmed presence of intact and monodisperse CPMV particles (diameter ∼30 nm, Fig. 1F).

The endotoxin in the pooled batch was 136 EU/mg, and was not expected to cause problems with the *in vivo* study conducted in rats due to the significantly (1000–10,000-fold) lower sensitivity of rodents to the toxic effects of endotoxin as compared to humans and other endotoxin-sensitive species [30], [31]. While we did not measure endotoxin in the plasma of CPMV-treated animals, we performed a theoretical estimation of what the maximum plasma endotoxin concentration might be when the current CPMV batch is administered to animals: the CPMV dose of 20 mg/kg, is equivalent to the endotoxin dose of 2720 EU/kg (or 20 mg/kg x 136 EU/mg). Considering an average body weight of a female Sprague Dawley rat of 250 g, this endotoxin dose is equivalent to 680 EU per rat and would result in a plasma endotoxin concentration of 34 EU/mL or 3.4 ng/mL (i.e., 680 EU/20 mL, where 20 mL is 8 % of the average body weight, and 1 EU=100 pg). The endotoxin concentration in the blood of healthy laboratory rats is 2–3 pg/mL [32]; that in healthy humans is 10–20 pg/mL [33]. The 3.4 ng/mL, which would be the maximum endotoxin dose achieved in our study, is comparable to the blood endotoxin concentrations in humans with certain pathological conditions [33] and is well below the concentrations (12.5 μg/mL at 1 mg/kg dose of LPS) that were reported earlier to produce no detectable toxicity in rats [31]. This estimation applies to i.v. administered CPMV. In the s.c. administered CPMV group, this estimation would apply only if the entire injected dose distributed to the systemic circulation; the plasma levels of endotoxin administered with CPMV upon s.c. injection is expected to be even lower. Therefore, endotoxin levels measured in the CPMV batch used for this in vivo study were not expected to cause acute toxicity in animals. Finally, β-glucan levels were assessed in several lots, and levels ranged from 303 to 1943 pg/mg CPMV. The detected beta-glucans were not expected to cause safety concerns when CPMV is used at the intended therapeutic dose because they would result in plasma beta-glucan levels not exceeding those normally present in the human blood from dietary sources [34].

### 3.2 *In vivo* toxicology

A repeat-dose toxicity study of CPMV was conducted in female Sprague Dawley rats. Rats were treated weekly with 5 mg fixed dose (∼20 mg/kg) CPMV or vehicle, s.c. or i.v., for three consecutive weeks. The s.c. route was chosen because it mimics the intended clinical route of administration, i.e. intratumorally. While our previous data indicates minimal leaching of CPMV after intratumoral administration [28], we included the i.v. group to mimic potential leaching and “worst case” systemic CPMV exposure. Rats were euthanized 24 h post-dose in the main study groups (acute toxicity group) or 14 days post-dose in the recovery groups. Recovery groups were used to assess whether an observable toxicity is partially or completely reversible after a dosing phase [35]. Untreated control animals were also included. At study termination, animals were subjected to complete necropsy, including gross description, body and organ weights, hematology and clinical chemistry, and histopathology of select organs.

### 3.3 In-life results

**Fig. 2:**
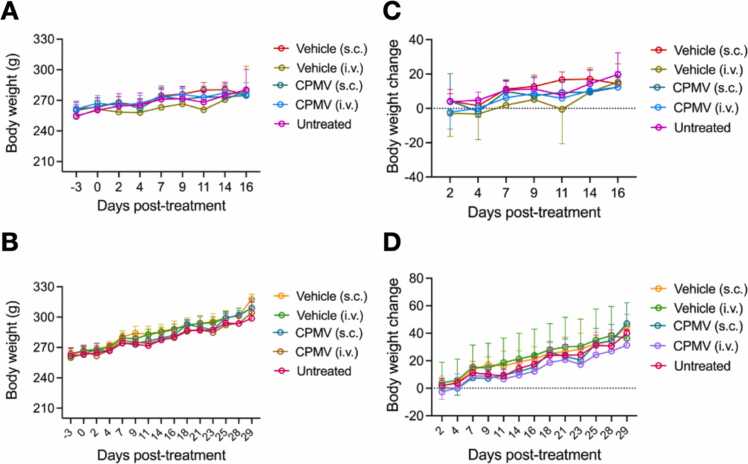
(A) Main and (B) recovery study absolute body weight. Absolute body weight is presented by treatment group over the main and recovery study periods. Data is presented as the mean ± SE (n = 7). (C) Main and (D) recovery study body weight change. Body weight change for each study day evaluated is displayed by treatment group. Data is presented as the mean ± SD (n = 7). No significant differences in body weight change were observed between treated and control groups (ANOVA with Dunnett’s test, p<0.05).

There were no deaths or abnormal clinical observations noted during the study period, and all animals reached their scheduled euthanasia times. Similar body weight gains were observed for treatment and control groups over the main and recovery study periods (Fig. 2, Tables S2 and S3).

### 3.4 Necropsy results

#### 3.4.1 Clinical chemistry parameters

Clinical chemistry parameters, such as electrolyte balance, and surrogate endpoints of carbohydrate metabolism, liver and pancreatic function were evaluated to determine possible CPMV biological effects (Table 3, Table 4, Table 5, Table 6). Statistically significant findings, including differences in amylase, phosphate, sodium, albumin, total protein, globulin and albumin/globulin (A/G) ratio, were variable in presentation (ANOVA with Dunnett’s test, p<0.05). These differences were either modest or within historical ranges (Charles River - Clinical Laboratory Parameters for the CD Rat, March 2006, www.criver.com) and unlikely to have biological significance, with the exception of increased globulin and decreased A/G ratio which appears to be related to the immunostimulatory mode of action of CPMV. The decreased A/G ratio was most pronounced in the group receiving CPMV s.c. and can be explained by presence of antigen presenting cells (APCs) including dendritic cells (DCs) in the subcutaneous space.

**Table 3:** s.c. main group clinical chemistry parameters. Clinical chemistry parameters are displayed by treatment group. Data is presented as the mean + SD (n = 7).

**s.c. Main Group**										
**Treatment**		**Untreated**			**Saline**			**5 mg (∼20 mg/kg) CPMV**		
Number of Animals		5			5			5		
**Electrolyte balance**										
Calcium	mg/dL	13.5	+	0.7	13.4	+	0.5	13.3	+	0.6
Phosphate	mg/dL	10.9*	+	1.0	8.4	+	1.2	9.9	+	0.8
Potassium	mmol/L	6	+	1	6.1	+	1.1	6.8	+	0.6
Sodium	mmol/L	147	+	2	147	+	3	147	+	2
**Carbohydrate metabolism**										
Glucose	mg/dL	352	+	52	373	+	84	301	+	63
**Pancreatic function**										
Amylase	u/L	740	+	99	795	+	76	545^*,**^	+	80
**Liver function:** **A) hepatobiliary**										
Total Bilirubin	mg/dL	0.3	+	0.0	0.3	+	0.0	0.3	+	0.0
**Liver function:** **B) hepatocellular**										
Alanine Aminotransferase	u/L	65	+	18	75	+	16	57	+	12
**Kidney function**										
Creatinine	mg/dL	0.3	+	0.1	0.3	+	0.1	0.3	+	0.1
Urea Nitrogen	mg/dL	15	+	2	17	+	2	14	+	2
**Others**										
Albumin (A)	g/dL	6.0	+	0.3	6.3	+	0.3	5.1^*,**^	+	0.3
Alkaline Phosphatase	u/L	175	+	21	181	+	58	150	+	37
Globulin (G)	g/dL	1.7	+	0.4	1.7	+	0.2	2.8^*,**^	+	0.3
A/G Ratio		3.8	+	1.2	3.7	+	0.4	1.9^*,**^	+	0.3
Total Protein		7.7	+	0.6	8.0	+	0.4	7.8	+	0.4

*Significantly different than untreated main group, ANOVA with Dunnett’s test, p< 0.05

**Significantly different than s.c. vehicle main group, ANOVA with Dunnett’s test, p< 0.05

**Table 4:** i.v. main group clinical chemistry parameters. Clinical chemistry parameters are displayed by treatment group. Data is presented as the mean + SD (n = 7).

**i.v. Main Group**										
**Treatment**		**Untreated**			**Saline**			**5 mg (∼20 mg/kg) CPMV**		
Number of Animals		5			5			5		
**Electrolyte balance**										
Calcium	mg/dL	13.5	+	0.7	13.5	+	0.6	13.0	+	0.4
Phosphate	mg/dL	10.9	+	1.0	9.8	+	0.8	9.1*	+	0.7
Potassium	mmol/L	6.5	+	1.3	6.4	+	0.7	6.1	+	0.8
Sodium	mmol/L	147	+	2	148	+	1	145^**^	+	2
**Carbohydrate metabolism**										
Glucose	mg/dL	352	+	52	361	+	89	317	+	57
**Pancreatic function**										
Amylase	u/L	740	+	99	750	+	124	503^*,**^	+	49
**Liver function:** **A) hepatobiliary**										
Total Bilirubin	mg/dL	0.3	+	0.0	0.3	+	0.0	0.3	+	0.0
**Liver function:** **B) hepatocellular**										
Alanine Aminotransferase	u/L	65	+	18	71	+	13	52	+	7
**Kidney function**										
Creatinine	mg/dL	0.3	+	0.1	0.4	+	0.1	0.4	+	0.0
Urea Nitrogen	mg/dL	15	+	2	15	+	2	16	+	2
**Others**										
Albumin (A)	g/dL	6.0	+	0.3	6.1	+	0.3	5.4^*,**^	+	0.2
Alkaline Phosphatase	u/L	175	+	21	210	+	64	172	+	48
Globulin (G)	g/dL	1.7	+	0.4	1.6	+	0.2	1.9	+	0.2
A/G Ratio		3.8	+	1.2	3.9	+	0.5	2.8^*,**^	+	0.3
Total Protein		7.7	+	0.6	7.6	+	0.4	7.3	+	0.3

*Significantly different than untreated main group, ANOVA with Dunnett’s test, p< 0.05

**Significantly different than i.v. vehicle main group, ANOVA with Dunnett’s test, p< 0.05

**Table 5:** s.c. recovery group clinical chemistry parameters. Clinical chemistry parameters are displayed by treatment group. Data is presented as the mean + SD (n = 7).

**s.c. Recovery Group**										
**Treatment**		**Untreated**			**Saline**			**5 mg (∼20 mg/kg)** **CPMV**		
Number of Animals		5			5			5		
**Electrolyte balance**										
Calcium	mg/dL	13.3	+	0.8	13.3	+	0.4	13.2	+	0.5
Phosphate	mg/dL	9.7	+	0.9	8.2*	+	0.7	10.3^**^	+	0.6
Potassium	mmol/L	5.8	+	1.1	6.0	+	0.7	6.0	+	1.1
Sodium	mmol/L	147	+	2	146	+	2	146	+	2
**Carbohydrate metabolism**										
Glucose	mg/dL	318	+	64	351	+	69	300	+	48
**Pancreatic function**										
Amylase	u/L	724	+	54	766	+	105	790	+	88
**Liver function:** **A) hepatobiliary**										
Total Bilirubin	mg/dL	0.3	+	0.0	0.3	+	0.0	0.3	+	0.0
**Liver function:** **B) hepatocellular**										
Alanine Aminotransferase	u/L	66	+	14	68	+	10	60	+	11
**Kidney function**										
Creatinine	mg/dL	0.2	+	0.0	0.3	+	0.1	0.3	+	0.1
Urea Nitrogen	mg/dL	15	+	2	17	+	3	16	+	3
**Others**										
Albumin (A)	g/dL	6.2	+	0.5	6.1	+	0.5	6.3	+	0.5
Alkaline Phosphatase	u/L	138	+	31	180	+	40	135	+	16
Globulin (G)	g/dL	1.3	+	0.2	1.7	+	0.2	8.4^*,**^	+	0.5
A/G Ratio		4.8	+	0.8	3.6	+	0.5	3.1*	+	0.4
Total Protein		7.5	+	0.5	7.7	+	0.5	2.1*	+	0.2

*Significantly different than untreated recovery group, ANOVA with Dunnett’s test, p< 0.05

**Significantly different than SC vehicle recovery group, ANOVA with Dunnett’s test, p< 0.05

**Table 6:** i.v. recovery group clinical chemistry parameters. Clinical chemistry parameters are displayed by treatment group. Data is presented as the mean + SD (n = 7).

**i.v. Recovery Group**										
**Treatment**		**Untreated**			**Saline**			**5 mg (∼20 mg/kg)** **CPMV**		
Number of Animals		5			5			5		
**Electrolyte balance**										
Calcium	mg/dL	13.3	+	0.8	13.0	+	0.6	13.4	+	0.5
Phosphate	mg/dL	9.7	+	0.9	9.2	+	1.5	10.3	+	0.9
Potassium	mmol/L	5.8	+	1.1	6.4	+	1.1	6.1	+	0.6
Sodium	mmol/L	147	+	2	147	+	2	145.1	+	1.2
**Carbohydrate metabolism**										
Glucose	mg/dL	318	+	64	320	+	107	334.57	+	77.46
**Pancreatic function**										
Amylase	u/L	724	+	54	678	+	76	812	+	75
**Liver function:** **A) hepatobiliary**										
Total Bilirubin	mg/dL	0.3	+	0.0	0.3	+	0.1	0.3	+	0.1
**Liver function:** **B) hepatocellular**										
Alanine Aminotransferase	u/L	66	+	14	75	+	4	57	+	7
**Kidney function**										
Creatinine	mg/dL	0.2	+	0.0	0.3	+	0.0	0.2	+	0.1
Urea Nitrogen	mg/dL	15	+	2	15	+	2	16	+	1
**Others**										
Albumin (A)	g/dL	6.2	+	0.5	6.0	+	0.3	6.0	+	0.3
Alkaline Phosphatase	u/L	138	+	31	162	+	39	136	+	33
Globulin (G)	g/dL	1.3	+	0.2	1.6	+	0.1	1.9*	+	0.2
A/G Ratio		4.8	+	0.8	3.9	+	0.4	3.2*	+	0.3
Total Protein		7.5	+	0.5	7.6	+	0.4	7.9	+	0.4

*Significantly different than untreated recovery group, ANOVA with Dunnett’s test, p< 0.05

#### 3.4.2 Hematology parameters

Bloodwork included complete blood counts (CBC) with confirmatory blood smears at the time of necropsy. Statistically significant changes in red blood cell distribution width, red blood cells, hemoglobin, hematocrit, white blood cells, neutrophils, monocytes, reticulocytes, and platelets were observed for treatment groups in comparison to controls (Table 7, Table 8, Table 9, Table 10) (ANOVA with Dunnett’s test, p<0.05Dunnett). The changes in erythrocyte parameters, including increased red blood cell distribution width and reticulocyte count, and decreased red blood cells, hemoglobin, and decreased hematocrit seen in both main and recovery s.c. and i.v. treated groups were modest and within historical values for the strain, sex, and age (Charles River - Clinical Laboratory Parameters for the CD Rat, March 2006, www.criver.com), but potentially biologically meaningful. Notably, these erythrocyte parameters did not recover in the recovery groups. Decreased platelets (thrombocytopenia) in the i.v. main group was dramatic in comparison to control values, but within the historic range, and recovery was observed in the recovery group. These changes in erythrocytes and platelets may reflect removal by the spleen which was enlarged.

**Table 7:** s.c. main group hematology parameters. Hematology parameters are displayed by treatment group. Data is presented as the mean ± SD (n = 7).

**s.c. Main Group**										
**Treatment**		**Untreated**			**Saline**			**5 mg (∼20 mg/kg)** **CPMV**		
Number of Animals		5			5			5		
**Leukocytes**										
WBC	K/uL	10.61	+	2.71	10.14	+	1.58	16.97^*,**^	+	3.37
NE	K/uL	0.97	+	0.54	0.81	+	0.18	3.72^*,**^	+	1.15
LY	K/uL	9.11	+	2.25	8.48	+	1.06	10.20	+	3.17
MO	K/uL	0.51	+	0.18	0.83	+	0.49	3.03^*,**^	+	0.77
EO	K/uL	0.02	+	0.01	0.02	+	0.01	0.02	+	0.01
BA	K/uL	0	+	0	0	+	0	0	+	0
**Erythrocytes**										
RBC	M/mL	7.96	+	0.59	8.53	+	0.66	7.50^*,**^	+	0.37
Hb	g/dL	14.7	+	1.1	15.4	+	1.0	13.6^*,**^	+	0.5
HCT	%	43.2	+	3.3	46.3	+	3.3	40.6^*,**^	+	1.6
MCV	fL	54.3	+	1.6	54.3	+	1.6	54.2	+	1.6
MCH	pg	18.4	+	0.6	18.1*	+	0.6	18.1	+	0.4
MCHC	g/dL	34.0	+	0.3	33.4	+	1.1	33.5	+	0.8
RDW	%	12.5	+	0.4	12.2	+	0.2	13.9^*,**^	+	0.4
Retics	(M/uL)	2.0	+	3.1	0.9	+	0.1	3.1	+	6.1
**Thrombocytes**										
PLT	K/mL	987	+	144	1032	+	117	999	+	162
MPV	fL	8.0	+	0.2	8.2	+	0.2	8.2	+	0.3

*Significantly different than untreated main group, ANOVA with Dunnett’s test, p< 0.05

**Significantly different than s.c. vehicle main group, ANOVA with Dunnett’s test, p< 0.05

**Table 8:** i.v. main group hematology parameters. Hematology parameters are displayed by treatment group. Data is presented as the mean ± SD (n = 7).

**i.v. Main Group**										
**Treatment**		**Untreated**			**Saline**			**5 mg (∼20 mg/kg)** **CPMV**		
Number of Animals		5			5			5		
**Leukocytes**										
WBC	K/uL	10.61	+	2.71	10.50	+	2.97	12.08	+	2.46
NE	K/uL	0.97	+	0.54	0.96	+	0.53	2.62^*,**^	+	0.75
LY	K/uL	9.11	+	2.25	8.95	+	2.26	8.52	+	1.91
MO	K/uL	0.51	+	0.18	0.58	+	0.28	0.92	+	0.29
EO	K/uL	0.02	+	0.01	0.03	+	0.02	0.02	+	0.01
BA	K/uL	0	+	0	0	+	0	0	+	0
**Erythrocytes**										
RBC	M/mL	7.96	+	0.59	8.33	+	0.54	7.66	+	0.38
Hb	g/dL	14.7	+	1.1	14.7	+	1.0	13.7	+	0.6
HCT	%	43.2	+	3.3	44.5	+	2.7	40.6^*,**^	+	1.4
MCV	fL	54.3	+	1.6	53.4	+	1.5	53.1	+	1.4
MCH	pg	18.4	+	0.6	17.6	+	0.6	17.9	+	0.5
MCHC	g/dL	34.0	+	0.3	32.9	+	0.7	33.7	+	0.7
RDW	%	12.5	+	0.4	12.2	+	0.5	13.5^*,**^	+	0.5
Retics	(M/uL)	2.0	+	3.1	0.8	+	0.1	22.0^*,**^	+	28.0
**Thrombocytes**										
PLT	K/mL	987	+	144	1039	+	63	427^*,**^	+	70
MPV	fL	8.0	+	0.2	8.2	+	0.1	7.9	+	0.2

*Significantly different than untreated main group, ANOVA with Dunnett’s test, p< 0.05

**Significantly different than i.v. vehicle main group, ANOVA with Dunnett’s test, p< 0.05

**Table 9:** s.c. recovery group hematology parameters. Hematology parameters are displayed by treatment group. Data is presented as the mean ± SD (n = 7).

**s.c. Recovery Group**										
**Treatment**		**Untreated**			**Saline**			**5 mg (∼20 mg/kg)** **CPMV**		
Number of Animals		7			7			7		
**Leukocytes**										
WBC	K/uL	10.10	+	1.40	9.65	+	2.61	11.88	+	2.61
NE	K/uL	0.93	+	0.27	0.75	+	0.28	1.39	+	0.46
LY	K/uL	8.73	+	1.20	8.24	+	2.30	9.87	+	2.07
MO	K/uL	0.42	+	0.17	0.64	+	0.25	0.60	+	0.24
EO	K/uL	0.02	+	0.01	0.02	+	0.01	0.02	+	0.01
BA	K/uL	0	+	0	0	+	0	0	+	0
**Erythrocytes**										
RBC	M/mL	7.68	+	0.39	7.78	+	0.36	7.11	+	2.6
Hb	g/dL	14.3	+	0.9	14.5	+	0.5	12.9	+	0.5
HCT	%	39.5	+	2.0	41.8	+	1.3	37.0	+	2.1
MCV	fL	51.5	+	1.8	53.7	+	1.9	45.8^**^	+	0.2
MCH	pg	18.6	+	0.9	18.7	+	0.4	16.0	+	0.0
MCHC	g/dL	36.1	+	0.6	34.8*	+	1.0	30.8	+	0
RDW	%	12.1	+	0.3	12.4	+	0.6	11.1^*,**^	+	3.3
Retics	(M/uL)	5.0	+	6.8	1.8	+	2.5	1.9	+	1.6
**Thrombocytes**										
PLT	K/mL	1083	+	164	973	+	53	1110	+	63
MPV	fL	8.2	+	0.4	8.0	+	0.1	7.9	+	0.4

*Significantly different than untreated recovery group, ANOVA with Dunnett’s test, p< 0.05

**Significantly different than s.c. vehicle recovery group, ANOVA with Dunnett’s test, p< 0.05

**Table 10:** i.v. recovery group hematology parameters. Hematology parameters are displayed by treatment group. Data is presented as the mean ± SD (n = 7).

**i.v. Recovery Group**										
**Treatment**		**Untreated**			**Saline**			**5 mg (∼20 mg/kg)** **CPMV**		
Number of Animals		7			7			7		
**Leukocytes**										
WBC	K/uL	10.10	+	1.40	10.44	+	1.80	13.17	+	1.62
NE	K/uL	0.93	+	0.27	1.01	+	0.39	1.30	+	0.16
LY	K/uL	8.73	+	1.20	8.94	+	1.79	11.29	+	1.48
MO	K/uL	0.42	+	0.17	0.47	+	0.10	0.54	+	0.23
EO	K/uL	0.02	+	0.01	0.02	+	0.02	0.03	+	0.02
BA	K/uL	0	+	0	0	+	0	0	+	0
**Erythrocytes**										
RBC	M/mL	7.68	+	0.39	8.14	+	0.80	7.25^**^	+	0.22
Hb	g/dL	14.3	+	0.9	14.8	+	1.2	13.7	+	0.3
HCT	%	39.5	+	2.0	42.3	+	4.0	38.5^**^	+	0.9
MCV	fL	51.5	+	1.8	51.9	+	2.0	53.0	+	1.7
MCH	pg	18.6	+	0.9	18.3	+	0.6	18.9	+	0.3
MCHC	g/dL	36.1	+	0.6	35.1	+	0.9	35.7	+	0.7
RDW	%	12.1	+	0.3	12.5	+	0.3	14.4^*,**^	+	0.5
Retics	(M/uL)	5.0	+	6.8	0.9	+	0.3	1.7	+	2.4
**Thrombocytes**										
PLT	K/mL	1083	+	164	1138	+	137	1098	+	103
MPV	fL	8.2	+	0.4	8.1	+	0.2	8.1	+	0.4

*Significantly different than untreated recovery group, ANOVA with Dunnett’s test, p< 0.05

**Significantly different than i.v. vehicle recovery group, ANOVA with Dunnett’s test, p < 0.05

Increases in white blood cells (leukocytosis), neutrophils (neutrophilia), and monocytes (monocytosis) were above historic values and support the immunostimulatory properties of the CPMV. These effects were more pronounced in the CPMV s.c. treated animals than the i.v. group, and resolution of these changes in parameters was observed in the recovery group. The observed changes in immune cell populations are consistent with CPMV’s mechanism of action: in tumor mouse models, we demonstrated that intratumoral administration activates, expands, and recruits innate immune cells: particularly M1 macrophages, N1 neutrophils, dendritic cells (DCs) and Natural Killer (NK) cells – in tumor models this would prime anti-tumor immunity, in healthy or tumor-bearing animals, this immune activation leads also to adaptive anti-immunity against CPMV [9], [13], [36], [37], [38].

#### 3.4.3 Organ weights

**Fig. 3:**
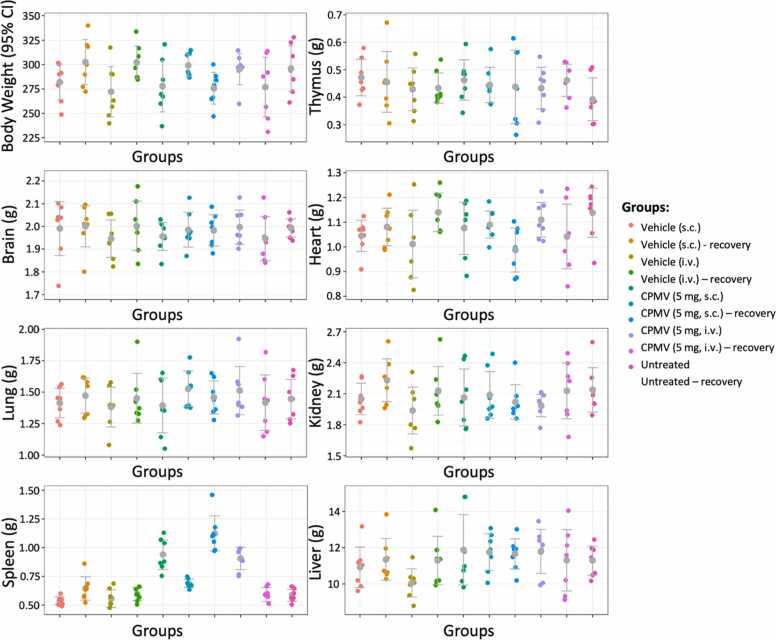
Relative organ weights separated by each group and group average is represented in grey with 95 % confidence interval. Please see Tables S4 and S5 for analysis of organ weight statistical differences.

Statistically significant increases in spleen weight values for both the i.v. and s.c. groups and liver weight for the i.v. group were observed for main study treatment groups in comparison to control groups (ANOVA with Dunnett’s test, p<0.05). These changes recovered in the s.c. recovery group but not the i.v. recovery group (Fig. 3, Tables S4+S5). No other statistically significant organ weight changes were identified.

### 3.5 Gross pathology

The gross lesions observed in main and recovery animal groups are summarized in 2.1, 2.2, 2.3, Table 1, Table 2, 2.4, 2.5, 2.6, 3.1, 3.2, 3.3, 3.4, 3.4.1, Table 3, Table 4, Table 5, Table 6, 3.4.2, Table 7, 3.4.3, 3.5, Appendix A. Gross lesions were identified inconsistently in control and treated animals. However, enlargement of lymph nodes (mesenteric, mediastinal, inguinal, axillary) and spleen was more prevalent in treated animals and correlated with histological changes in some cases. All other gross necropsy observations were considered spontaneous or incidental because they are common findings in laboratory animals this animal model, occurred at a low incidence, or were randomly distributed across both treated and control groups.

**Fig. 4:**
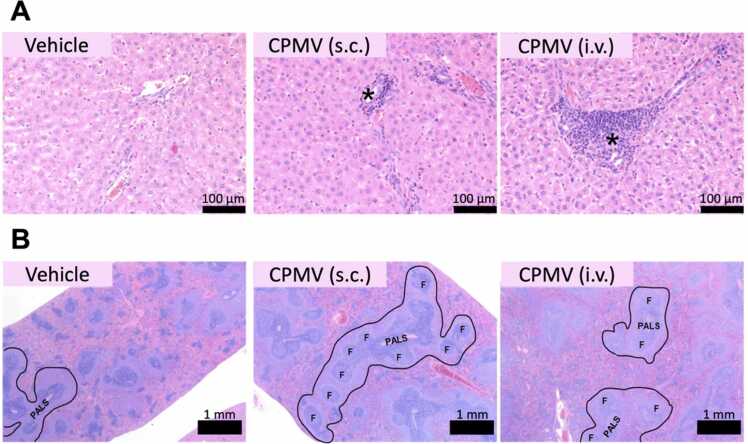
(A) Representative Liver Histopathology. Portal areas are often mildly to moderately expanded by increased lymphocytes with fewer plasma cells, neutrophils, or histiocytic cells. In addition, randomly scattered throughout the hepatic lobules were multifocal, typically mild accumulations of mononuclear cells, including histiocytic cells and lymphocytes. Extramedullary hematopoiesis was observed rarely (B) Representative Spleen Histopathology. The periarteriolar lymphoid sheathes are often prominent with and often contained germinal centers and follicular structures. Prominent marginal zones were observed for all rats in all groups. Extramedullary hematopoiesis was observed in the red pulp, along with increased histiocytic cells. Splenic congestion was observed.

Tables S8 and S9 and Fig. 4 summarize the histopathological findings observed in the main and recovery groups. Select tissues identified by gross, hematological, or clinical chemistry abnormalities (spleen, liver, femur bone marrow, and lymph node) were evaluated by a board-certified veterinary pathologist for histopathological analysis.

In the main and recovery study, a statistically significant increase in hepatic periportal lymphocytic infiltrate was observed in the i.v. group in comparison to controls (Kruskal-Wallis ANOVA with multiple comparisons test, p < 0.05). A trend toward increased lymph node hyperplasia consisting of increased nodules often containing germinal centers was observed in both the main study i.v. and s.c. treatment groups but did not reach statistical significance. No significant histologic findings are observed in bone marrow, regardless of the treatment group.

Hepatic portal areas are often mildly to moderately expanded by increased lymphocytes with fewer plasma cells, neutrophils, or histiocytic cells (Fig. 4A). In addition, randomly scattered throughout the hepatic lobules were multifocal, typically mild accumulations of mononuclear cells, including histiocytic cells and lymphocytes. Extramedullary hematopoiesis was observed rarely. The periportal inflammation observed in the liver was most pronounced in the i.v. main group and was less prominent in the recovery group; this change was also observed in the main s.c. group.

Statistically significant increases in splenic lymphoid apoptosis and hyperplasia, extramedullary hematopoiesis were observed in the main study i.v. and s.c. treatment group in comparison to controls, and the splenic hyperplasia remained in the i.v. recovery group (Kruskal-Wallis ANOVA with multiple comparisons test, p<0.05). The periarteriolar lymphoid sheathes were often prominent and often contained germinal centers and follicular structures (Fig. 4B). Splenic congestion was observed, as well as prominent marginal zones for all rats in all groups. Extramedullary hematopoiesis was observed in the red pulp, along with increased histiocytic cells.

The remaining histological lesions identified were of mild severity and considered spontaneous or incidental because they occurred at low incidence, were randomly distributed across groups (including controls) or interpreted as background changes commonly seen in rats of this gender and age, not being considered related to CPMV treatment.

## 4 Discussion

The objective of this study was to evaluate the repeat-dose toxicity of CPMV in female Sprague Dawley rats. Administration of CPMV resulted in reactive immune stimulation, observed grossly and histologically in the spleen and lymph nodes with evidence of increased lymphocytic infiltrates in the liver. Enlargement of lymph nodes and spleen was more prevalent in treated animals and correlated with histological changes such as hyperplasia, in some cases, supporting the immunostimulatory mechanism of action of CPMV. Beyond its immunodulatory mechanism of action leading to anti-tumor immunity, CPMV is immunogenic resulting in cell- [13] and antibody-mediated responses to it [18]. In previous work, we have not observed any CPMV-specific IgE responses and, therefore, immediate type I hypersensitivity reactions (true allergy) are unlikely. While the presence of CPMV-specific IgM and IgG could trigger complement activation and complement-mediated anaphylactoid reactions, this has not been observed in any prior study, including *in vitro* complement activation analysis using human plasma [24] and canine cancer trials [14]. Of note, complement activation by nanoparticles in dogs was reported to be stronger than in humans with a strong predictive value for human complement [39]; therefore, the canine studies provide further reassurance that the risk of anaphylactoid reactions in human is low. In fact, pre-existing anti-CPMV antibodies in healthy human blood samples has been observed (50 % in a 50 patient pool [28]); taking together, these data suggest that the risk of anaphylactoid reactions and true allergy to CPMV adjuvant is low. In terms of CPMV’s immunogenicity and prevalence of anti-drug-antibodies (ADAs), while CPMV-specific IgM and IgG have been observed in tumor mouse models and canine cancer patients, they were not neutralizing. Instead, these antibodies enhanced the intended therapeutic immunostimulation by promoting CPMV uptake through Fc receptors expressed on the surface of immune cells [18].

Changes in bloodwork were also noted and these were also are consistent with a reactive immune processes. Overall, the changes are mild and appear to be largely reversible and non-adverse. The decrease in red blood cell, which was mild, was persistent in recovery groups; however, given no significant histologic findings in the bone marrow, this change is likely non-adverse and a longer timeline is required to fully evaluate red blood cell changes.

Neither a maximum tolerated dose (MTD) nor a no-observable adverse effect level (NOAEL) was established in this study. With regards to dosing and future clinical trials: our standard dose for treatment of mouse tumors is 0.1 mg CPMV per treatment per ∼20 mm^2^ tumor area or 5 μg CPMV per mm^2^ tumor. Canine patients received doses of 0.2–1 mg CPMV per treatment per 3–15 cm^2^ or ∼0.5 μg CPMV/mm^2^ tumor. Given the safety profile and lack of MTD finding at the 5 mg dose, it is possible and expected that an MTD cannot be achieved by intratumoral dosing. For example, 5 mg CPMV per 3 cm^2^ tumor equates to a 0.06 mg/kg dose (per 70 kg patient). Dosing beyond 30 mg per tumor (0.4 mg/kg) is likely not feasible due to limits in injection volumes (1/3 of the size of the tumor) and CPMV concentration limits (50 mg/mL), and a dose < 1 mg/kg is not likely to manifest toxicity. If an MTD cannot be determined, we will determine a recommended dose, which either could be defined based on a biomarker, i.e. a certain level of type I interferons, in plasma or based on injection volume limitations. Overall, data indicate a good safety profile of the CPMV cancer immunotherapy candidate. Follow-up, IND-enabling GLP toxicology and pharmacology studies should consider higher concentrations of CPMV and longer time frame for the recovery group.

## 5 Conclusions

This study evaluated the pharmacology and safety of cowpea mosaic virus (CPMV) as a novel cancer immunotherapy candidate. The repeat-dose toxicity assessment in Sprague Dawley rats revealed no treatment-related mortality or significant clinical abnormalities. The observed changes in albumin/globulin ratio, leukocytosis, neutrophilia, monocytosis, and lymphoid hyperplasia are consistent with an immunostimulatory response, supporting the proposed mechanism of action for CPMV. Other statistically significant clinical chemistry, hematology, and histopathology findings were mild, within historical ranges for the model, and not considered biologically significant.

Importantly, neither a maximum tolerated dose (MTD) nor a no-observable adverse effect level (NOAEL) was established, indicating a favorable safety profile within the tested dose range. These findings provide critical preclinical evidence supporting the continued clinical development of CPMV as a promising immunotherapy approach for cancer. Future studies should explore dose escalation, long-term effects, and potential immune-related adverse events in additional preclinical and clinical settings.

## CRediT authorship contribution statement

**Steinmetz Nicole:** Writing – review & editing, Writing – original draft, Supervision, Project administration, Investigation, Funding acquisition, Conceptualization. **Dobrovolskaia Marina A.:** Writing – review & editing, Writing – original draft, Project administration, Investigation, Funding acquisition. **Neun Barry W.:** Writing – review & editing, Investigation. **Edmondson Elijah:** Writing – review & editing, Investigation. **Gatus Jamie:** Writing – review & editing, Investigation. **de Oliveira Jessica Fernanda Affonso:** Writing – review & editing, Writing – original draft, Investigation. **Stern Stephan T.:** Writing – review & editing, Writing – original draft, Investigation, Conceptualization.

## Funding sources

This work was supported in part by 10.13039/100000002NIH grants R01 CA253615 and R01 CA274640 (to N.F.S.), the 10.13039/100000048American Cancer Society – F.M. Kirby Foundation Inc. – Mission Boost Grant, MBGI-23–1030244-01-MBG (to N.F.S.). N.F.S. acknowledges support through the Shaughnessy Family Fund for Nano-ImmunoEngineering (nanoIE) at UCSD. The project has been funded in part (S.T.S., B.W.N, E.E, and M.A.D.) with federal funds from the 10.13039/100000054National Cancer Institute, 10.13039/100000002National Institutes of Health, under contract 75N91010D00024. The content of this publication does not necessarily reflect the views or policies of the Department of Health and Human Services, nor does mention of trade names, commercial products, or organizations imply endorsement by the US Government.

## Declaration of Competing Interest

The authors declare the following financial interests/personal relationships which may be considered as potential competing interests: Nicole Steinmetz reports a relationship with PlantiosX Inc that includes: equity or stocks. Nicole Steinmetz has patent issued to Case Western Reserve University & UC San Diego. If there are other authors, they declare that they have no known competing financial interests or personal relationships that could have appeared to influence the work reported in this paper.

## Data Availability

Data will be made available on request.
