# Supplementary material for: Preclinical SC and IV repeat-dose toxicology of a cowpea mosaic virus – A cancer immunotherapy candidate
Source: Toxicol Rep. 2025 Apr 7;14:102022. doi: 10.1016/j.toxrep.2025.102022 (PMC12019203; doi:10.1016/j.toxrep.2025.102022)
Supplement: Supplementary file 1 — Supplementary material [file mmc1.pdf]

Supporting Information for

Preclinical SC and IV repeat-dose toxicology of a cowpea mosaic virus – a cancer immunotherapy candidate

*Stephan T. Stern[1], Jessica Fernanda Affonso de Oliveira[2-4], Jamie Gatus[2-4], Elijah Edmondson[6], Barry W. Neun[1], Marina A. Dobrovolskaia[1], Nicole F. Steinmetz[2-5,7-10]**

[1]Nanotechnology Characterization Lab, Cancer Research Technology Program, Frederick National Laboratory for Cancer Research sponsored by the National Cancer Institute, Frederick, MD, United States

[2]Aiiso Yufeng Li Family Department of Chemical and Nano Engineering, University of California San Diego, 9500 Gilman Dr., La Jolla, CA 92093, USA

[3]Shu and K.C. Chien and Peter Farrell Collaboratory, University of California San Diego, 9500 Gilman Dr., La Jolla, CA 92093, USA

[4]Center for Nano-ImmunoEngineering, University of California San Diego, 9500 Gilman Dr., La Jolla, CA 92093, USA

[5]Department of Bioengineering, University of California San Diego, 9500 Gilman Dr., La Jolla, CA 92093, USA

[6]Molecular Histopathology Laboratory, Laboratory of Animal Sciences Program, Frederick National Laboratory for Cancer Research sponsored by the National Cancer Institute, 8560 Progress Drive, Frederick, MD 21701, USA.

[7]Department of Radiology, University of California San Diego, 9500 Gilman Dr., La Jolla, CA 92093, USA

[8]Institute for Materials Discovery and Design, University of California San Diego, 9500 Gilman Dr., La Jolla, CA 92093, USA

[9]Moores Cancer Center, University of California San Diego, 9500 Gilman Dr., La Jolla, CA 92093, USA

[9]Center for Engineering in Cancer, Institute of Engineering Medicine, University of California San Diego, 9500 Gilman Dr., La Jolla, CA 92093, USA

* Corresponding authors: nsteinmetz@ucsd.edu

Table S1. Randomization Report

Randomized by body weight (g)										
**Body Weight (g)**										
**Group**	**F**	**Dose**	**M**	**T**	**Mean**	**StdDev**	**SEM**	**Min**	**Max**	**% Diff**
Vehicle (Main group) – s.c.	7	Once a week/3x	0	7	261.43	17.39	6.57	227.00	280.00	0.47
Vehicle (Recovery group) – s.c.	7		0	7	261.86	14.86	5.62	237.00	277.00	0.63
Vehicle (Main group) – i.v.	7		0	7	253.86	26.60	10.06	215.00	284.00	2.44
Vehicle (Recovery group) – i.v.	7		0	7	259.86	10.12	3.83	245.00	272.00	0.14
5 mg (~20 mg/kg) CPMV (Main group) – s.c.	7		0	7	260.14	23.40	8.84	222.00	289.00	0.03
5 mg (~20 mg/kg) CPMV (Recovery group) – s.c.	7		0	7	263.71	13.50	5.10	241.00	279.00	1.35
5 mg (~20 mg/kg) CPMV (Main group) – i.v.	7		0	7	261.43	20.65	7.80	233.00	292.00	0.47
5 mg (~20 mg/kg) CPMV (Recovery group) – i.v.	7		0	7	263.71	15.92	6.02	237.00	285.00	1.35
Untreated (Main group)	7		0	7	254.57	29.53	11.16	220.00	294.00	2.17
Untreated (Recovery group)	7		0	7	261.57	21.39	8.09	239.00	289.00	0.52
Brown-Forsythe:			W =	1.4485	P =	0.1884				
ANOVA:			F =	0.1993	P =	0.9934				

Table S2. Main Study Body Weight Change. Body weight change for each study day evaluated is displayed by treatment group. Data is presented as the mean ± SD (n = 7). *NB:* no significant differences in body weight change were observed between treated and control groups ($p<0.05$, ANOVA with Dunnett's test).

Day	SC, Vehicle	IV, Vehicle	SC, 5 mg CPMV	IV, 5 mg CPMV	Untreated
2	4.14 ± 4.41	-2.86 ± 13.47	4.29 ± 16.04	-2.14 ± 9.92	3.86 ± 7.17
4	1.57 ± 3.31	-3.29 ± 15.00	-1.86 ± 3.29	0.00 ± 4.51	4.71 ± 4.68
7	11.14 ± 4.74	1.86 ± 14.90	9.86 ± 5.87	6.14 ± 5.73	10.57 ± 5.71
9	12.71 ± 5.77	5.14 ± 14.35	7.29 ± 5.38	8.57 ± 9.74	11.29 ± 6.45
11	16.71 ± 4.54	-0.57 ± 20.04	9.29 ± 6.05	5.86 ± 4.53	7.71 ± 8.98
14	17.00 ± 5.60	9.43 ± 14.36	9.43 ± 6.70	10.29 ± 5.59	14.29 ± 7.72
16	14.33 ± 4.51	15.33 ± 10.69	12.33 ± 5.51	12.33 ± 9.07	19.67 ± 12.66

Table S3. Recovery Study Body Weight Change. Body weight change for each study day evaluated is displayed by treatment group. Data is presented as the mean ± SD (n = 7). *NB:* no significant differences in body weight change were observed between treated and control groups ($p<0.05$, ANOVA with Dunnett's test).

Day	SC, Vehicle - Rec	IV, Vehicle - Rec	SC, 5 mg CPMV - Rec	IV, 5 mg CPMV - Rec	Untreated - Rec
2	1.57 ± 7.21	3.86 ± 15.09	0.43 ± 5.91	-2.71 ± 5.28	2.00 ± 5.29
4	4.43 ± 2.99	5.86 ± 15.40	-0.14 ± 4.88	0.17 ± 10.21	3.71 ± 3.04
7	13.43 ± 6.00	15.43 ± 16.05	7.57 ± 5.26	9.00 ± 7.77	11.29 ± 4.79
9	17.14 ± 5.64	14.71 ± 17.96	7.14 ± 5.64	8.67 ± 7.17	10.00 ± 3.61
11	16.29 ± 10.64	18.71 ± 17.63	9.57 ± 7.93	6.67 ± 5.43	8.86 ± 4.91
14	19.00 ± 8.27	21.29 ± 18.66	12.14 ± 7.58	9.50 ± 9.63	14.43 ± 5.91
16	21.29 ± 8.98	23.86 ± 19.06	15.43 ± 9.47	12.33 ± 7.37	17.57 ± 5.41
18	25.43 ± 7.68	28.00 ± 18.95	26.00 ± 7.98	18.67 ± 9.05	24.00 ± 5.42
21	26.57 ± 10.44	30.00 ± 21.60	23.00 ± 12.78	20.83 ± 9.22	24.00 ± 7.64
23	28.71 ± 11.35	30.43 ± 19.89	20.57 ± 8.42	17.33 ± 11.29	24.29 ± 6.13
25	32.14 ± 8.69	35.00 ± 22.55	32.43 ± 11.13	24.33 ± 8.02	31.29 ± 7.36
28	34.29 ± 8.16	38.29 ± 21.29	34.71 ± 11.67	27.00 ± 9.32	30.71 ± 9.52
29	45.33 ± 8.50	36.33 ± 5.77	47.00 ± 15.13	31.33 ± 3.79	40.00 ± 2.65

Table S4. Main Study Organ Weights. The organ weights are displayed as absolute values and % body weight, by treatment group.

Treatment		s.c. Vehicle	i.v. Vehicle	s.c. 5 mg CPMV	i.v. 5 mg CPMV	Untreated
**Number of Animals**		**7**	**7**	**7**	**7**	**7**
**Body Weight**						
Absolute Weight	Gram	282.08 ± 19.95	272.29 ± 27.88	277.77 ± 28.25	275.67 ± 17.64	276.92 ± 32.89
**Brain**						
Absolute Weight	Gram	1.99 ± 0.13	1.95 ± 0.09	1.96 ± 0.07	1.98 ± 0.07	1.95 ± 0.11
Per body weight	%	0.71 ± 0.06	0.72 ± 0.07	0.71 ± 0.07	0.72 ± 0.03	0.71 ± 0.06
**Heart**						
Absolute Weight	Gram	1.04 ± 0.07	1.01 ± 0.15	1.08 ± 0.11	0.99 ± 0.10	1.04 ± 0.14
Per body weight	%	0.37 ± 0.02	0.37 ± 0.03	0.39 ± 0.02	0.36 ± 0.02	0.38 ± 0.03
**Kidney**						
Absolute Weight	Gram	2.05 ± 0.16	1.94 ± 0.25	2.06 ± 0.30	2.02 ± 0.18	2.13 ± 0.29
Per body weight	%	0.73 ± 0.09	0.72 ± 0.08	0.74 ± 0.05	0.73 ± 0.06	0.77 ± 0.04
**Liver**						
Absolute Weight	Gram	10.91 ± 1.20	10.05 ± 0.84	11.87 ± 2.10	11.65 ± 0.90	11.30 ± 1.83
Per body weight	%	3.87 ± 0.34	3.70 ± 0.19	4.25 ± 0.39	4.23*** ± 0.24	4.06 ± 0.23
**Lung**						
Absolute Weight	Gram	1.41 ± 0.13	1.38 ± 0.17	1.39 ± 0.24	1.46 ± 0.14	1.42 ± 0.24
Per body weight	%	0.50 ± 0.05	0.51 ± 0.05	0.50 ± 0.06	0.53 ± 0.07	0.51 ± 0.05
**Spleen**						
Absolute Weight	Gram	0.54 ± 0.04	0.56 ± 0.08	0.94*,** ± 0.14	1.12*,*** ± 0.17	0.59 ± 0.07
Per body weight	%	0.19 ± 0.02	0.20 ± 0.01	0.34*,** ± 0.03	0.41*,*** ± 0.08	0.22 ± 0.03
**Thymus**						
Absolute Weight	Gram	0.54 ± 0.07	0.43 ± 0.08	0.46 ± 0.08	0.44 ± 0.14	0.46 ± 0.06
Per body weight	%	0.19 ± 0.02	0.16 ± 0.03	0.17 ± 0.03	0.16 ± 0.05	0.17 ± 0.03

Data is presented as the mean ± SD (n = 7).

*Significantly different than untreated main group, Dunnett test, $p \leq 0.05$

**Significantly different than s.c. vehicle main group, Dunnett test, $p \leq 0.05$

***Significantly different than i.v. vehicle main group, Dunnett test, $p \leq 0.05$

Table S5. Recovery Study Organ Weights. The organ weights are displayed as absolute values and % body weight, by treatment group. Data is presented as the mean ± SD (n = 7).

Treatment		s.c. Vehicle - Rec	i.v. Vehicle - Rec	s.c. 5mg CPMV - Rec	i.v. 5mg CPMV - Rec	Untreated - Rec
**Number of Animals**		**7**	**7**	**7**	**7**	**7**
**Body Weight**						
Absolute Weight	Gram	302.61 ± 24.78	302.18 ± 18.43	299.22 ± 12.16	295.62 ± 17.27	296.02 ± 25.23
**Brain**						
Absolute Weight	Gram	2.00 ± 0.10	2.00 ± 0.12	1.99 ± 0.08	2.00 ± 0.08	1.99 ± 0.04
Per body weight	%	0.67 ± 0.05	0.66 ± 0.03	0.66 ± 0.04	0.68 ± 0.05	0.68 ± 0.06
**Heart**						
Absolute Weight	Gram	1.08 ± 0.08	1.14 ± 0.08	1.09 ± 0.06	1.11 ± 0.08	1.14 ± 0.11
Per body weight	%	0.36 ± 0.04	0.38 ± 0.03	0.36 ± 0.01	0.38 ± 0.03	0.39 ± 0.03
**Kidney**						
Absolute Weight	Gram	2.23 ± 0.22	2.13 ± 0.25	2.09 ± 0.24	1.98 ± 0.12	2.14 ± 0.23
Per body weight	%	0.74 ± 0.05	0.70 ± 0.06	0.70 ± 0.06	0.67 ± 0.01	0.73 ± 0.08
**Liver**						
Absolute Weight	Gram	11.34 ± 1.25	11.29 ± 1.44	11.75 ± 1.09	11.79 ± 1.32	11.29 ± 0.87
Per body weight	%	3.75 ± 0.26	3.73 ± 0.29	3.92 ± 0.26	3.98 ± 0.33	3.82 ± 0.14
**Lung**						
Absolute Weight	Gram	1.47 ± 0.15	1.45 ± 0.21	1.53 ± 0.15	1.51 ± 0.21	1.45 ± 0.17
Per body weight	%	0.49 ± 0.04	0.48 ± 0.05	0.51 ± 0.04	0.51 ± 0.05	0.49 ± 0.04
**Spleen**						
Absolute Weight	Gram	0.65 ± 0.11	0.60 ± 0.06	0.69 ± 0.04	0.91*,*** ± 0.11	0.59 ± 0.06
Per body weight	%	0.21 ± 0.04	0.20 ±	0.23 ± 0.01	0.31*,*** ± 0.03	0.20 ± 0.02
**Thymus**						
Absolute Weight	Gram	0.46 ± 0.12	0.43 ± 0.06	0.44 ± 0.07	0.43 ± 0.08	0.39 ± 0.08
Per body weight	%	0.15 ± 0.03	0.14 ± 0.01	0.15 ± 0.02	0.15 ± 0.03	0.13 ± 0.02

*Significantly different than untreated recovery group, Dunnett test, p≤0.05
**Significantly different than SC vehicle recovery group, Dunnett test, p≤0.05
***Significantly different than IV vehicle recovery group, Dunnett test, p≤0.05

Table S6. Main Group Gross Pathology. Findings are displayed by treatment group.

MAIN GROUP						
	Females	Treatment Group				
	DOSE GROUP	s.c. Vehicle	i.v. Vehicle	s.c. 5 mg CPMV	i.v. 5 mg CPMV	Untreated
ORGAN/ TISSUE	Lesions					
Ovary	# of Organs	7	7	7	7	7
	Both Discolored Red	2	5	1	1	
Lung	# of Organs	7	7	7	7	7
	Diaphragmatic foci Round Tan	1				
Kidney	# of Organs	7	7	7	7	7
	Both foci Round Dark	1	1			
	Nodule Round Red Firm		1			
	Left Focus Irregular Dark				1	
Uterus	# of Organs	7	7	7	7	7
	Both horns Dilation	1		1		1
	Both horns Discolored					1
LN, Mesenteric	# of Organs	7	7	7	7	7
	Both Enlarged Oval Tan				2	
Liver	# of Organs	7	7	7	7	7
	Median Nodule Round Tan Soft			1		
	All lobes Enlarged Red			1		
	All lobes Reticular pattern				1	
LN, Inguinal	# of Organs	7	7	7	7	7
	Enlarged Oval Tan			3	1	
LN, Lumbar	# of Organs	7	7	7	7	7
	Enlarged Oval Tan				1	
LN, Axillary	# of Organs	7	7	7	7	7
	Enlarged Oval Tan Firm			4	1	
LN, Mediastinal	# of Organs	7	7	7	7	7
	Enlarged Oval Tan Firm			2	1	
LN, Mandibular	# of Organs	7	7	7	7	7
	Enlarged Oval Tan Firm			1	1	2
LN, Deep Cervical	# of Organs	7	7	7	7	7
	Enlarged Oval Tan Firm			2	2	
LN, Iliac	# of Organs	7	7	7	7	7
	Enlarged Oval Tan Firm			1	1	
Spleen	# of Organs	7	7	7	7	7
	Enlarged			2	1	
	Deformity			1		
Kidney	# of Organs	7	7	7	7	7
	Left Nodule Round Tan					1

Table S7. Recovery Group Gross Pathology. Findings are displayed by treatment group.

RECOVERY GROUP						
	Females	Treatment Group				
	DOSE GROUP	s.c. Vehicle - Rec	i.v. Vehicle - Rec	s.c. 5 mg CPMV - Rec	i.v. 5 mg CPMV - Rec	Untreated - Rec
ORGAN/ TISSUE	Lesions					
Ovary	# of Organs	7	7	7	7	7
	Both Discolored Red			3	1	
	Both Discolored Mottled	1			1	
Lung	# of Organs	7	7	7	7	7
	Left foci Round White		1			
	All lobes Discolored Mottled				1	
Kidney	# of Organs	7	7	7	7	7
	Both Nodule Round White Firm		1			
	Both Discolored Mottled		1			
Liver	# of Organs	7	7	7	7	7
	Left Deformity Red		1			
	All lobes Enlarged Red					
Adrenal	# of Organs	7	7	7	7	7
	Both Discolored Dark	1	1			
Nerve	# of Organs	7	7	7	7	7
	Trigeminal Enlarged Tan		1			
LN, Inguinal	# of Organs	7	7	7	7	7
	Enlarged Oval Tan Firm			3		
LN, Inguinal	# of Organs	7	7	7	7	7
	Enlarged Oval Tan Firm			1		
LN, Axillary	# of Organs	7	7	7	7	7
	Enlarged Oval Tan Firm			1		
LN, Mediastinal	# of Organs	7	7	7	7	7
	Enlarged Oval Tan Firm			1		
Pancreas	# of Organs	7	7	7	7	7
	Discolored Pale				1	
Spleen	# of Organs	7	7	7	7	7
	Granular				1	

Table S8. Main Group Histopathology Findings. Findings are displayed by treatment group.

	Females		Treatment Group				
	DOSE GROUP		s.c. Vehicle	i.v. Vehicle	s.c. 5 mg CPMV	i.v. 5 mg CPMV	Untreated
ORGAN/ TISSUE	Lesions	Grade					
Femur, bone marrow	# of Organs		7	7	7	7	7
Liver	# of Organs		7	7	7	7	7
	Extramedullary hematopoiesis	+			1		
	Lymphocytic infiltrate, periportal	+	1	1	5		3
		++			2		
		+++				7*,***	
	Mononuclear cell infiltrate	+	7	7	4	7	7
		++			2		
LN, Mesenteric	# of Organs		7	7	7	7	7
	Histiocytosis	+	2	1	1		3
		++	5	6	6	5	4
	Apoptosis	++				1	
	Hyperplasia, lymphoid	+			1	1	
		++			1	1	
		+++				1	
Spleen	# of Organs		7	7	7	7	7
	Apoptosis, lymphoid	+			3	2*,***	
		++				5	
	Congestion	+			1		
	Extramedullary hematopoiesis, increased	+	1	2	3*,**	4	2
		++			3	2	
		+++			1		
	Hyperplasia, lymphoid	+	4	4			3
		++		1	6*	3*	
		+++			1	4	

*Significantly different than untreated main group, Kruskal-Wallis ANOVA with multiple comparisons test ($p \leq 0.05$)
**Significantly different than SC vehicle main group, Kruskal-Wallis ANOVA with multiple comparisons test ($p \leq 0.05$)
***Significantly different than IV vehicle main group, Kruskal-Wallis ANOVA with multiple comparisons test ($p \leq 0.05$)

Table S9. Recovery Group Histopathology Findings. Findings are displayed by treatment group.

	Females		Treatment Group				
	DOSE GROUP		s.c. Vehicle - Rec	i.v. Vehicle - Rec	s.c. 5 mg CPMV - Rec	i.v. 5 mg CPMV - Rec	Untreated - Rec
ORGAN/ TISSUE	Lesions	Grade					
Femur, bone marrow	# of Organs		7	7	7	7	7
Liver	# of Organs		7	7	7	7	7
	Lymphocytic infiltrate, periportal	+	4	5	6		2
		++			1	3[*,***]	
		+++				4	
	Mononuclear cell infiltrate	+	6	7	7	5	7
		++				2	
LN, Mesenteric	# of Organs		7	7	7	7	7
	Histiocytosis	+	2	3	2	3	1
		++	3	4	3	4	4
	Hyperplasia, lymphoid	+				2	
Spleen	# of Organs		7	7	7	7	7
	Congestion	+		2			3
		++					1
	Extramedullary hematopoiesis, increased	+	2	2	5	5	3
	Histiocytosis	+			1		
	Hyperplasia, lymphoid	+	2	3			3
		++	1		7	4[*,***]	1
		+++				3	

*Significantly different than untreated recovery group, Kruskal-Wallis ANOVA with multiple comparisons test ($p \leq 0.05$)
**Significantly different than SC vehicle recovery group, Kruskal-Wallis ANOVA with multiple comparisons test ($p \leq 0.05$)
***Significantly different than IV vehicle recovery group, Kruskal-Wallis ANOVA with multiple comparisons test ($p \leq 0.05$)
